# Transport of Ca^2+^ and Ca^2+^-Dependent Permeability Transition in the Liver and Heart Mitochondria of Rats with Different Tolerance to Acute Hypoxia

**DOI:** 10.3390/biom10010114

**Published:** 2020-01-09

**Authors:** Konstantin N. Belosludtsev, Mikhail V. Dubinin, Eugeny Yu. Talanov, Vlada S. Starinets, Kirill S. Tenkov, Nadezhda M. Zakharova, Natalia V. Belosludtseva

**Affiliations:** 1 Institute of Theoretical and Experimental Biophysics, Russian Academy of Sciences, Institutskaya 3, Pushchino, 142290 Moscow Region, Russia; 2 Department of Biochemistry, Cell Biology and Microbiology, Mari State University, pl. Lenina 1, Yoshkar-Ola, 424001 Mari El, Russiakirill.tenkove@gmail.com (K.S.T.); 3 Institute of Cell Biophysics, Russian Academy of Sciences, PSCBR RAS, Institutskaya 3, Pushchino, 142290 Moscow Region, Russia

**Keywords:** hypoxia, resistance to hypoxia, mitochondria, mitochondrial calcium transport, mitochondrial calcium uniporter complex, mitochondrial Ca^2+^-induced permeability transition pore, cyclophilin D, ATP synthase

## Abstract

The work examines the kinetic parameters of Ca^2+^ uptake via the mitochondrial calcium uniporter complex (MCUC) and the opening of the Ca^2+^-dependent permeability transition pore (MPT pore) in the liver and heart mitochondria of rats with high resistance (HR) and low resistance (LR) to acute hypoxia. We found that the rate of Ca^2+^ uptake by mitochondria of the liver and heart in HR rats is higher than that in LR rats, which is associated with a higher level of the channel-forming subunit MCU in liver mitochondria of HR rats and a lower content of the dominant-negative channel subunit MCUb in heart mitochondria of HR rats. It was shown that the liver mitochondria of HR rats are more resistant to the induction of the MPT pore than those of LR rats (the calcium retention capacity of liver mitochondria of HR rats was found to be 1.3 times greater than that of LR rats). These data correlate with the fact that the level of F_0_F_1_-ATP synthase, a possible structural element of the MPT pore, in the liver mitochondria of HR rats is lower than in LR rats. In heart mitochondria of rats of the two phenotypes, no statistically significant difference in the formation of the MPT pore was revealed. The paper discusses how changes in the expression of the MCUC subunits and the putative components of the MPT pore can affect Ca^2+^ homeostasis of mitochondria in animals with originally different tolerance to hypoxia and in hypoxia-induced tissue injury.

## 1. Introduction

Hypoxia/ischemia is a widespread phenomenon that occurs both in conditions of oxygen deficiency in the environment and in various pathologies as a result of a decrease in oxygen delivery to the cell to a level insufficient to maintain its functions and structure. Hypoxic conditions in the body are observed in ischemic and reperfusion injuries of organs, hemodynamic disorders, coronary insufficiency, blood loss, hemorrhagic shock, arterial hypertension, pulmonary insufficiency, systemic inflammatory response syndrome, traumatic shock, exposure to adverse environmental factors, extreme conditions, and other stressful effects.

The main intracellular targets of hypoxia of various etiologies are mitochondria and aerobic energy metabolism [1,2]. Hypoxia alters mitochondrial dynamics and morphology and provokes mitochondrial dysfunction, which can lead to a drop in the ATP synthesis, an increase in the production of reactive oxygen species, and other adverse events, including the disturbance of ionic, mainly Ca^2+^, homeostasis. Excessive accumulation of Ca^2+^ in cytoplasm and mitochondria in hypoxia and subsequent reoxygenation can finally initiate cell injury and death via the opening of the Ca^2+^-dependent mitochondrial permeability transition pore (MPT pore) and the subsequent release of proapoptotic proteins from organelles [3].

As a major large-capacity buffer system of Ca^2+^ ions, mitochondria are involved in the regulation of intracellular Ca^2+^ homeostasis. Changes in mitochondrial [Ca^2+^] modulate key cellular processes, ranging from aerobic metabolism (through Ca^2+^-sensitive dehydrogenases, and enzymes of the Krebs cycle [4]) to the release of proapoptotic factors [5,6,7,8], as well as local modulation of the activity of channels and enzymes [9,10].

In recent studies, it has been found that mitochondria contain several proteins involved in mitochondrial Ca^2+^ uptake: mitochondrial Ca^2+^ uniporter complex (MCUC), RaM (the rapid mode of Ca^2+^ uptake), Letm1 (leucine zipper and EF-hand-containing transmembrane protein 1), mitochondrial ryanodine receptor type 1, and uncoupling proteins [5,11]. Among them, the MCUC is believed to be the main calcium transport system, which mediates the electrophoretic influx of the ion into mitochondria. This multiprotein complex is composed of the pore-forming and calcium-conducting subunit MCU (mitochondrial calcium uniporter subunit), its paralogue MCUb (mitochondrial calcium uniporter dominant negative beta subunit), and regulatory subunits: the small membrane-spanning protein EMRE (essential MCU regulatory subunit), the peripheral membrane proteins acting as gatekeepers MICU1 and MICU2 (mitochondrial calcium uptake proteins 1 and 2), and MCUR1 (MCU regulator protein 1) [5,6]. Variations in the ratio of the subunits of MCUC determine the dynamics of mitochondrial Ca^2+^ uptake in different tissues and pathophysiological states. For example, a decrease in the expression of MICU1 in the heart tissue correlates with the lowered activation threshold of Ca^2+^ influx and the decreased calcium capacity of heart mitochondria in comparison with those of liver mitochondria [12]. MCUb mRNA is highly expressed in the heart and minimally expressed in skeletal muscle, which can be the cause of variations in the tissue-dependent mitochondrial Ca^2+^ uptake: the Ca^2+^ influx into skeletal muscle mitochondria is significantly greater than that into cardiac ones [13].

Excessive accumulation of Ca^2+^ in mitochondria leads to the opening of the Ca^2+^-dependent mitochondrial permeability transition pore (MPT pore), which can be a key step in the mechanism of the activation of programmed cell death or, under certain conditions, can serve as an additional nonspecific calcium release pathway. The molecular structure of the MPT pore is as yet not clearly established. The MPT pore complex is considered to be a multiprotein mega-channel, which includes proteins of the inner and outer mitochondrial membranes. Among possible candidates for the role of the channel-forming subunit of the MPT pore are considered a number of proteins of the inner mitochondrial membrane, including ATP synthase, adenine nucleotide translocator, and phosphate carrier. At the same time, the only protein that is currently claimed to be an integral part of the MPT pore is the regulatory protein cyclophilin D, which is able to interact with the above-mentioned proteins. Cyclophilin D is the target of the cyclic undecapeptide cyclosporin A (CsA), which desensitizes the MPT pore complex to calcium ions at nanomolar concentrations [5,14].

Studies on the role of mitochondrial Ca^2+^ transport systems in the pathogenesis of hypoxia have been ongoing for a long time. It was found that the pharmacological or genetic modulation of cyclophilin D leads to the inhibition of pore opening in mitochondria and increases the resistance of cell cultures and tissues to the hypoxic state. At the same time, the question of how the structure and operation of the mitochondrial Ca^2+^ transport system and the MPT pore can be regulated in hypoxia and hypoxic adaptation remains unclear. The answer to this question should be found when conducting studies on animals with different individual resistance to acute hypoxia [15,16,17]. It is known that the onset and severity of the acute hypoxia response of rats drastically differ within the population, and two opposite, extreme phenotypes of animals, with high resistance (HR) and low resistance (LR) to hypoxia, have significantly different “functional-metabolic profiles” [18]. Thus, we have shown earlier that the liver mitochondria of HR and LR rats have different resistance to the formation of Ca^2+^-dependent mitochondrial pores of two types, the MPT pore and the lipid pore induced by saturated long-chain fatty acids [19].

In the present work, we attempted to determine the structural and functional features of the mitochondrial Ca^2+^ transport multiprotein systems, the MCUC and the MPT pore, in liver and heart tissues of animals with different tolerance to oxygen shortage. The study demonstrates that: (1) the rate of accumulation of Ca^2+^ ions by mitochondria isolated from the liver and heart of HR rats is considerably higher than that of LR rats, which highly correlates changes observed in the content, the ratio, and mRNA level of the subunits of the MCUC. In HR rats, the levels of the pore-forming uniporter subunit, MCU, and the key regulatory uniporter subunit, MICU1, in liver mitochondria are significantly higher than in LR rats. The level of the dominant-negative channel subunit, MCUb, is lower in heart mitochondria of HR rats compared to those of LR rats; (2) the liver mitochondria of HR animals are more resistant to the induction of the MPT pore than those of LR animals. This difference can be associated with the lower level of ATP synthase, a protein considered to be the channel-forming component of the MPT pore, in liver tissue of HR animals. In heart mitochondria of rats of the two phenotypes, no difference in the formation of MPT pore was revealed.

## 2. Materials and Methods

### 2.1. Testing the Tolerance of Rats to Extreme Hypoxia Conditions

Experiments were conducted on Wistar male rats (220–250 g) with different baseline resistance to hypoxia, low-resistance (LR) and high-resistance (HR) rats. The resistance of rats was tested one month prior to the experiments using a special procedure [19]. Animals were placed into a hypoxic chamber and then tested for the ability to endure a critical life-incompatible O_2_ concentration. For this test, the concentration of oxygen in the chamber was reduced to 3.1% by its displacement with nitrogen gas, and the time of the onset of pathological breathing (apnea) (T_a_) was determined. This parameter characterizes the viability of animals under extreme hypoxic conditions and reflects the ability to fully mobilize the nonspecific protective functions responsible for the survival of the organism in the sublethal period. After registering T_a_, the hypoxic chamber was opened, and the normal posture and locomotor activity of the animals restored within 5–10 min. The T_a_ value was 1–2 min for LR rats and more than 10 min for HR rats. Each group of rats amounted to approximately 20% of the total number of animals tested (n = 30). An interval of one month after the procedure of testing the tolerance of rats to hypoxia conditions was chosen on the basis of earlier studies to eliminate the effect of hypoxic exposure and to identify the basal differences between two rat phenotypes [15,16,18]. After this interval, two extreme types of animals with different tolerance to acute oxygen deficiency exhibit characteristic features of the ultrastructure and functional activity of mitochondria of different organs [16,19].

The laboratory animals were treated in accordance with the European Convention for the Protection of Vertebrates used for experimental and other purposes (Strasbourg, 1986) and the principles of the Helsinki Declaration (2000). All the protocols were approved by the Institute of Theoretical and Experimental Biophysics RAS Ethics Committee (Order No. 173/k of 03.10.2011, Protocol No. 03/2019 of 05.03.2019).

### 2.2. Isolation of Rat Liver and Heart Mitochondria

Mitochondria were isolated from the liver and heart of Wistar rats by differential centrifugation as described earlier [19]. The homogenization buffer contained 210 mM mannitol, 70 mM sucrose, 1 mM EDTA, and 10 mM Hepes/KOH buffer, pH 7.4. Subsequent centrifugations were performed in the same buffer, except that, instead of EDTA, 100 μM EGTA was used. Final suspensions contained 70–80 mg of mitochondrial protein/mL and 25–35 mg of mitochondrial protein/mL (liver and heart mitochondria, respectively), as determined by the Lowry method [20].

### 2.3. Ca^2+^ Uptake by Mitochondria

The Ca^2+^ concentration in the incubation medium was monitored spectrophotometrically with an arsenazo III indicator at 675–685 nm using a plate reader Tecan Spark 10M (Tecan, Männedorf, Switzerland) at 25 °C under constant stirring. At a neutral pH, Ca^2+^ forms a complex with arsenazo III, which has a blue color. The color intensity is proportional to the concentration of Ca^2+^ in a buffer and can be measured spectrophotometrically. Mitochondria (0.4–0.5 mg of mitochondrial protein/mL) were suspended in an incubation medium containing 210 mM mannitol, 70 mM sucrose, 1 mM KH_2_PO_4_, 50 μM arsenazo III, 10 μM EGTA, and 10 mM HEPES-KOH (pH 7.4) and energized with 2.5 mM glutamate + 2.5 mM malate. After the addition of 50 μM CaCl_2_, the rate of Ca^2+^ uptake by mitochondria (nmol Ca^2+^ × min^−1^ × mg^−1^ of mitochondrial protein) was determined in the presence of 1 μM CsA. To determine the ability of mitochondria to retain Ca^2+^, 10 μM CaCl_2_ was added into the reaction medium successively, with an interval of ~90 s. After several additions, external [Ca^2+^] increased, indicating a massive release of the ion from the organelles due to the opening of the MPT pore. The amount of Ca^2+^ released upon permeability transition (defined as Ca^2+^ retention capacity) was used as a measure of the MPT pore opening probability.

### 2.4. Electrophoresis and Immunoblotting of Mitochondrial Proteins

To prepare samples for quantifying the levels of mitochondrial proteins, aliquots of native mitochondria (2 mg/mL) were solubilized in Laemmli buffer in Eppendorf tubes and heated for 3 min at 95 °C. Sample aliquots normalized by the protein concentration (10 µg of mitochondrial protein) were applied to the lanes and subjected to electrophoresis followed by Western blot analysis. Mitochondrial samples were separated by 12.5% SDS-PAGE and transferred to a 0.45 µm nitrocellulose membrane (Amersham, Munich, Germany). The proteins of PageRuler Prestained Protein Ladder (Thermo Scientific, Waltham, MA USA) were used as markers. After overnight blocking, the membrane was incubated with the appropriate primary antibody. The monoclonal rabbit anti-MCU (#14997), anti-CBARA/MICU1 (#12524), and anti-ANT2/SLC25A5 (#14671) antibodies were from Cell Signaling Technology Inc. (Danvers, MA, USA). The total OXPHOS Rodent WB Antibody Cocktail (#ab110413) containing the α-subunit of complex V (CV-ATP5A-55 kDa), anti-VDAC1 (#ab154856), and the polyclonal rabbit antibodies Anti-CCDC109B (#ab170715), anti-cyclophilin F (CypD) (#ab64935), and anti-ANT1 (#ab102032) were from Abcam (Cambridge, United Kingdom). The immunoreactivity was detected using the appropriate secondary antibody conjugated to horseradish peroxidase (#7074, Cell Signaling Technology Inc., Danvers, MA, USA). Peroxidase activity was detected with ECL chemiluminescence reagents (Pierce, Rockford, IL, USA). The relative levels of the detected proteins were visualized using an LI-COR system (LI-COR, Lincoln, NE, USA). Optical density measurements were performed using the LI-COR Image Studio software.

### 2.5. RNA Extraction, Reverse Transcription, and Quantitative Real-Time PCR

Total RNA was isolated from 100 mg of deep-frozen tissue samples (the liver or the heart) using an ExtractRNA kit (Eurogen, Moscow, Russia) in accordance with the protocol of the manufacturer. The resulting RNA preparation was treated with RNase-free DNase I (Thermo Scientific, Waltham, MA USA). The concentration of total RNA was measured spectrophotometrically using a Nanodrop ND-1000 spectrophotometer (ND Technologies, Fremont, CA, USA). Two micrograms of total RNA was taken for cDNA synthesis; reverse transcription was performed using an oligo(dT)15 primer and MMLV reverse transcriptase (Eurogen, Moscow, Russia) according to the manufacturer’s instructions. Real-time PCR was performed using a DTLite5 amplifier (DNA-Technology LLC, Moscow, Russia) using the qPCRmix-HS SYBR reaction mixture (Eurogen, Moscow, Russia). The selection and analysis of gene-specific primers were performed using Primer-BLAST [21] (the oligonucleotide sequences are presented below; see Table 1). The relative level of the expression of each gene was normalized by the level of mRNA of the cytoskeletal protein beta-actin (Actb), and a comparative C_T_ method was used to quantify the results [22].

### 2.6. Statistical Analysis

The data were analyzed using the GraphPad Prism 7 and Excel software and presented as mean (median) ± SEM of 3–5 experiments. Statistical differences between the means were determined by Mann–Whitney U test; *p* < 0.05 was considered to be statistically significant.

## 3. Results

### 3.1. Mitochondrial Ca^2+^ Uptake and the Features of Subunit Composition of the Mitochondrial Ca^2+^ Uniporter Complex in the Liver and Heart of Rats with Different Tolerance to Acute Hypoxia

**Figure 1:**
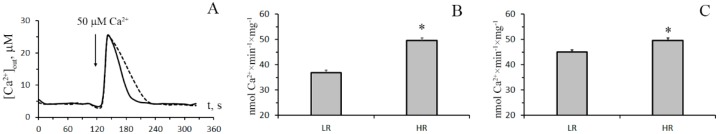
Ca^2+^ ion uptake by mitochondria of the liver and heart of hypoxia low-resistance (LR) and high-resistance (HR) rats. (**A**) The changes in the external concentration of Ca^2+^ ions in the incubation medium during their accumulation by the liver mitochondria of HR (the solid line) and LR (the dotted line) rats. The incubation medium contained 210 mM mannitol, 70 mM sucrose, 2.5 mM malate, 2.5 mM glutamate, 1 mM KH_2_PO_4_, 10 μM EGTA, 1 μM cyclosporin A, and 10 mM Hepes/KOH buffer (pH 7.4). Additions: rat liver mitochondria (0.4 mg/mL), 50 μM CaCl_2_. The typical traces of five independent experiments are presented. (**B**) The rates of Ca^2+^ uptake by liver mitochondria (shaded columns) of HR and LR rats. (**C**) The rates of Ca^2+^ uptake by heart mitochondria of HR and LR rats. Values are given as means ± SEM (n = 5). * The difference between HR and LR animals is statistically significant (*p* < 0.05).

In this work, we first analyzed the functional and structural features of the system of mitochondrial Ca^2+^ transport in the liver and the heart of animals with different tolerance to oxygen shortage, LR and HR rats. Figure 1A shows the kinetics of uptake of Ca^2+^ (50 μM) by the liver mitochondria of LR (dotted line) and HR rats (solid line) in the presence of CsA, which was necessary to block the possible opening of the MPT pore. One can see that the rate of Ca^2+^ influx into the liver mitochondria of HR rats is 1.3 times higher compared with that of LR rats (Figure 1B). The heart mitochondria of HR rats were found to accumulate Ca^2+^ ions also significantly faster than those of LR rats, although the difference is less pronounced than for liver mitochondria (about 10–15%) (Figure 1C).

Mitochondrial Ca^2+^ uptake is mediated by an electrogenic uniport, referred to as “Ca^2+^ uniporter”, a complex of proteins of the inner mitochondrial membrane, including the pore-forming subunit MCU and its dominant-negative form MCUb, and the regulatory subunits MICU1, MICU2, EMRE, and MCUR1. It is considered that the contents of MCU, MCUb, and MICU1 and their stoichiometry can predominantly regulate the mitochondrial Ca^2+^ transport in accordance with physiological needs [5,6,23]. Since the mitochondria of HR animals accumulate Ca^2+^ faster as compared to the organelles of LR rats, one can assume that the adaptation to hypoxic stress is associated with changes in the relative content of these subunits in the mitochondrial membrane. Therefore, we quantified the uniporter protein constituents and their mRNA level in the liver and cardiac muscle of rats depending on the baseline resistance of animals to hypoxia.

**Figure 2:**
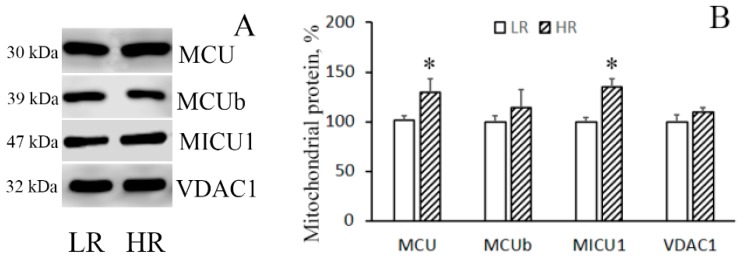

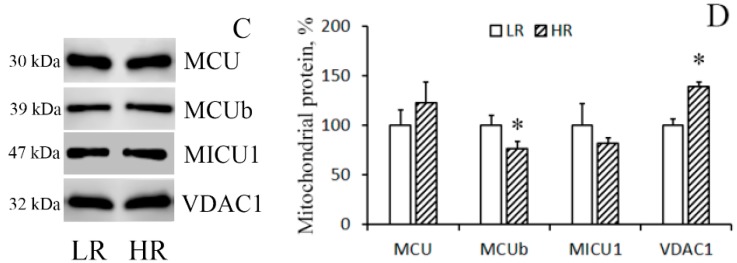
Levels of the subunits of MCUC and VDAC1 in liver (**A**,**B**) and heart (**C**,**D**) mitochondria of LR and HR rats. Western blot analysis of the members of the MCU protein family (MCU, MCUb, and MICU1) and VDAC1 in the liver (**A**) and heart (**C**) mitochondria of LR and HR rats. Summarized data of densitometric band analysis of these proteins in the liver (**B**) and heart (**D**) mitochondria of LR and HR rats. Values are given as means ± SEM (n = 3). * The difference between HR and LR animals is statistically significant (*p* < 0.05).

The immunoblotting of the members of the MCUC protein family of the mitochondria isolated from the liver of rats of two phenotypes reveals that in HR rats, the levels of MCU and MICU1 were higher than in LR rats by 1.3 and 1.35 times, respectively (Figure 2A,B), whereas no significant difference in the level of the dominant-negative uniporter subunit MCUb was observed. As for heart tissue, a comparative analysis of the protein level of the components of the MCUC showed that there was a significant decrease in the content of MCUb and a slight tendency to an increase in the content of MCU in the cardiac mitochondria of HR rats relative to those of LR animals (Figure 2C,D). VDAC1 participates in the formation of MAM (mitochondria-associated membranes) contacts, which are the main pathway of Ca^2+^ transfer from the endoplasmic reticulum to mitochondria [5,14]. One can see that the amount of VDAC1 in liver mitochondria did not differ. At the same time, the level of VDAC1 in heart mitochondria of HR rats was higher than that of LR rats.

**Figure 3:**
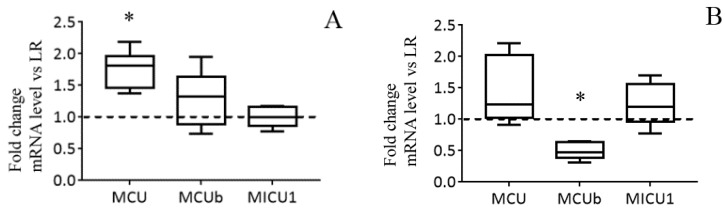
The mRNA levels of the subunits of MCUC in liver (**A**) and heart (**B**) of HR rats normalized to those of LR rats. The median and extreme values are presented (n = 8). The horizontal line is the mean value of the mRNA levels in LR rats. * The difference between HR and LR animals is statistically significant (*p* < 0.05).

The results of the real-time PCR analysis confirm the data obtained and also indicate that the changes occur at the level of transcription. The mRNA content of MCU in the liver of HR rats is significantly increased in comparison with that of LR rats. At the same time, the expression profile of MICU1 and MCUb in the liver of rats of two phenotypes does not differ (Figure 3A). So, the MCU/MCUb expression ratio grew from 4.6 in the liver of LR animals to 7.0 in the liver of HR rats (2^−ΔCt^ *MCU* = 0.014 ± 0.001 and 0.025 ± 0.001 in the liver tissue of LR and HR rats, respectively; 2^−ΔCt^ *MCUb* = 0. 003 ± 0.0005 and 0.0035 ± 0.00038 in LR and HR rats, respectively). Figure 3B demonstrates that the expression level of MCUb is significantly lower in the heart tissue of HR animals in comparison with that of LR animals. The MCU/MCUb expression ratio grew from 3.5 in the heart of LR animals to 8.8 in the heart of HR rats (2^−ΔCt^ *MCU* = 0.024 ± 0.005 and 0.034 ± 0.008 in the heart tissue of LR and HR rats, respectively; 2^−ΔCt^ *MCUb* = 0.007 ± 0.0007 and 0.0038 ± 0.0005 in LR and HR rats, respectively).

On the basis of the results obtained, one can conclude that an increase in the relative amount of the pore-forming subunit of MCUC, MCU, in liver mitochondria and a decrease in the dominant-negative subunit, MCUb, in heart mitochondria underlie the increased rate of mitochondrial Ca^2+^ uptake in HR rats relative to that in LR rats.

### 3.2. A Comparison of the Resistance of Mitochondria to the Opening of MPT Pore and of the Levels of Its Probable Molecular Components in the Liver and Heart of HR and LR Rats

**Figure 4:**
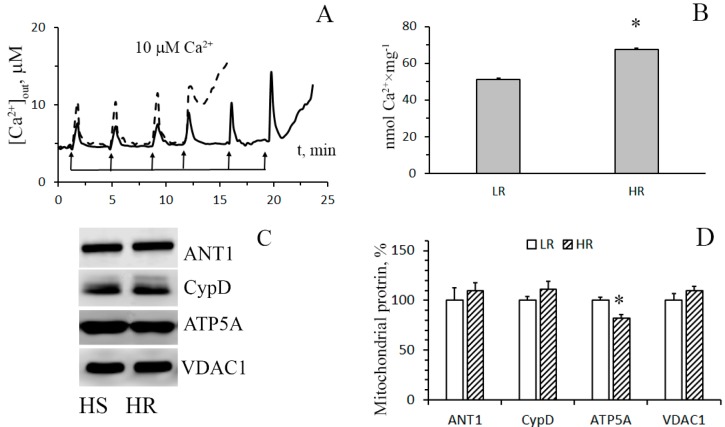
The parameters of formation and levels of putative protein components of the Ca^2+^-induced MPT pore in the liver mitochondria of HR and LR rats. (**A**) Changes in the external concentration of Ca^2+^ in a suspension of liver mitochondria of HR (the solid line) and LR (the dotted line) rats after sequential additions of 10 μM Ca^2+^. The incubation medium was the same as in Figure 1, with the exception of cyclosporin A. Additions: rat liver mitochondria (0.4 mg/mL). The figure shows traces of a typical experiment conducted at the same time on the same mitochondrial preparation. Similar results were obtained in five independent experiments. (**B**) Calcium retention capacity of liver mitochondria of LR and HR rats. The values are given as means ± SEM (n = 5). (**C**) Western blot analysis of MPT-related proteins: CypD, ANT1, ATP5A, and VDAC1 in liver mitochondria of LR and HR rats. (**D**) Summarized data on the relative contents of the MPT-related proteins. Values are given as means ± SEM (n = 3). * The difference between HR and LR animals is statistically significant (*p* < 0.05).

The pathophysiological phenomenon of the Ca^2+^-induced MTP pore opening is well known to be central to mitochondrial vital functions and can play a lethal role in many pathophysiological conditions including hypoxia cell injury. So, the next objective of our work was to examine whether the resistance of mitochondria to the opening of the MPT pore changes depending on the baseline tolerance of an animal to hypoxic condition. The mitochondrial Ca^2+^ retention capacity (CRC) is related to the threshold concentration of Ca^2+^ necessary for the pore to open. One of the ways to assess this parameter is to introduce Ca^2+^ into the suspension of mitochondria in small successive doses, and the number of such additions until the MPT pore is triggered will reflect the CRC of the organelles. Figure 4A shows the results of such experiments. It can be seen that the number of successive Ca^2+^ additions (and, therefore, the threshold pore-opening Ca^2+^ concentration) in the case of the liver mitochondria of HR rats was greater compared to the organelles of the liver of LR rats. The parameter of CRC of HR rat liver mitochondria increased 1.3-fold (Figure 4B). This implies that their resistance to the opening of the MPT pore would also be higher.

By now, the structure of the MPT pore, which is believed to be a mega-channel penetrating both the inner and outer mitochondrial membranes, has yet to be determined. Three proteins of the inner mitochondrial membrane, namely, adenylate translocator (ANT), ATP synthase, and cyclophilin D, are considered to be essential components of the pore complex [14,24]. To elucidate a possible molecular mechanism of the resistance of HR rat liver mitochondria to the induction of MPT pore opening, we have compared the levels of these proteins in the organelles of the liver of rats of two phenotypes.

**Figure 5:**
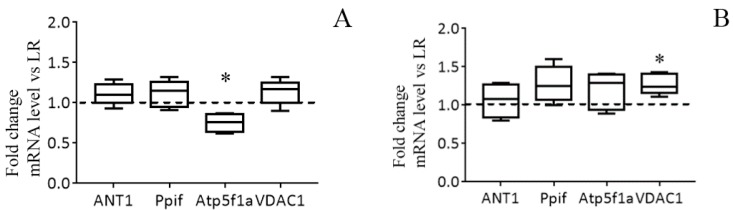
The mRNA levels of the MPT in liver (**A**) and heart (**B**) of HR rats normalized to those of LR rats. The median and extreme values are presented (n = 8). The horizontal line is the mean value of the mRNA levels in LR rats. * The difference between HR and LR animals is statistically significant (*p* < 0.05).

Figure 4C,D show immunoblots of cyclophilin D, ANT1, and α-subunit of ATP synthase of mitochondria from the liver of HR and LR rats. One can see that the amount of cyclophilin D and ANT1 in the mitochondria did not differ. The levels of mitochondrial ATP synthase in the liver of HR rats, on the other hand, were reduced, with the reduction being statistically significant. The results of the real-time PCR analysis confirm the data obtained and also indicate that the changes occur at the level of transcription. So, the mRNA content of *Atp5f1a* in the liver of HR rats is significantly decreased in comparison with that of LR rats. At the same time, the expression profile of ANT1 and cyclophilin D in the liver of rats of two phenotypes does not differ (Figure 5A). We suppose that the decrease in the content of ATP synthase can be the cause of the increased resistance of liver mitochondria of HR rats to the MPT pore opening.

**Figure 6:**
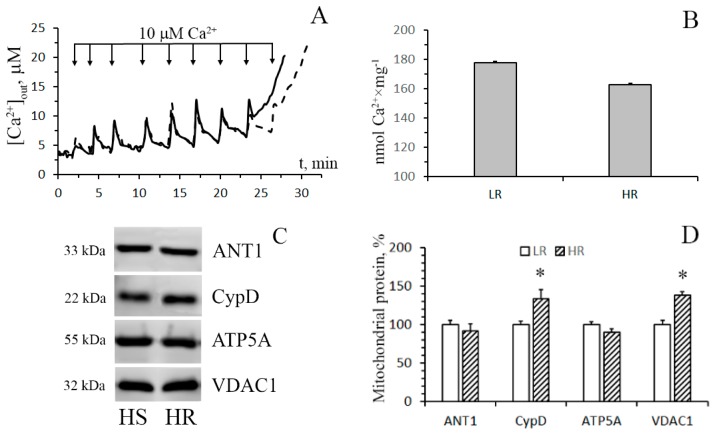
Induction of the Ca^2+^-induced MPT pore in the heart mitochondria of HR and LR rats. (**A**) Changes in the external concentration of Ca^2+^ in a suspension of heart mitochondria of HR (the solid line) and LR (the dotted line) rats after sequential additions of 10 μM Ca^2+^. The incubation medium was the same as in Figure 1, with the exception of cyclosporin A. Additions: heart mitochondria (0.4 mg/mL); 10 μM CaCl_2_. The figure shows traces of a typical experiment conducted at the same time on the same mitochondrial preparation. Similar results were obtained in five independent experiments. (**B**) Calcium retention capacity of heart mitochondria of LR and HR rats. The values are given as means ± SEM (n = 5). The medium composition and experimental conditions were as indicated in Figure 4A. (**C**) Western blot analysis of MPT-related proteins: CypD, ANT1, ATP5A, and VDAC1, in heart mitochondria of LR and HR rats. (**D**) Summarized data on the relative contents of the MPT-related proteins. Values are given as means ± SEM (n = 3). * The difference between HR and LR animals is statistically significant (*p* < 0.05).

Figure 6 shows the data of the estimation of maximal calcium capacity and Western blot analysis of the MPT-related proteins in mitochondria from the heart of HR and LR animals. In contrast to mitochondria from the liver tissue, cardiac mitochondria from rats of both groups display no statistically significant difference in the CRC and the content of ATP synthase and ANT1. At the same time, the levels of the regulatory protein, cyclophilin D, and VDAC1, which is assumed to form the channel of the MPT pore in the outer mitochondrial membrane, in heart mitochondria of HR rats were higher than those of LR rats. The results of real-time PCR analysis are consistent with the data of immunoblotting (Figure 5B).

## 4. Discussion

It is generally accepted that mitochondria are one of the main intracellular targets of hypoxia. Structural, biochemical, and functional abnormalities of mitochondria are widely believed to be important pathogenetic factors that underlie hypoxic or ischemic cell injury [1,18]. Apart from disordering mitochondrial ATP synthesis, network dynamics, and redox state, hypoxia also dysregulates the Ca^2+^ homeostasis of these organelles and the cell as a whole. The hypoxia-induced dramatic alterations in Ca^2+^ transport in mitochondria may not only be related to the decreased oxidative phosphorylation (OXPHOS), a metabolic shift toward glycolysis, and the development of oxidative stress, but also induce cell death pathways via mitochondrial Ca^2+^ overload and the formation of Ca^2+^-dependent MPT pore in the inner mitochondrial membrane, leading to the release of proapoptotic proteins into the cytosol [3]. Consequently, it has been proposed that mitochondrial Ca^2+^ transport pathways might be targets for protective intervention, as well as involved in the formation of the molecular mechanism of cell resistance to hypoxia and ischemia. The purpose of our work was to study in more detail the features of structure and operation of the Ca^2+^ transport systems in the mitochondria of the liver and heart of rats with originally different tolerance to acute hypoxia. 

The results obtained in this work indicate that mitochondria of HR and LR rats are characterized by fundamentally different genetically programmed rearrangements of the systems regulating Ca^2+^ homeostasis. In the case of HR rats, the rearrangements of the Ca^2+^ uniporter can increase the efficiency of Ca^2+^ accumulation by liver and heart mitochondria. At the same time, the organelles of the liver of HR rats become more resistant to the opening of the Ca^2+^-dependent MPT pore in comparison with those of LR rats. Along with the well-characterized distinction in the intensity of OXPHOS in mitochondria of the vital organs of LR and HR rats [18,25], these effects might contribute to the development of individual systemic resistance of animals to acute hypoxia. 

It is well known that individuals of an animal population differ in their tolerance to oxygen deficiency [15,16,17,25]. As shown earlier, animals that belong to two opposite, in regard to hypoxia tolerance, types (LR and HR) have essentially different “functional-metabolic” profiles, including the effectiveness of energy support of the organism, the regulation of central nervous cardiovascular systems, neurohumoral regulation, stress-activating and stress-limiting systems, the oxygen-transporting function of blood, and the state of membranes and receptors. LR animals are considered to have a weak type of the nervous system, increased excitability, and emotional reactivity. LR animals respond to hypoxia with agitation and high locomotor activity. In contrast, HR animals have reduced excitability and anxiety, milder aggressiveness, more pronounced internal inhibition, low sensitivity to any provocative factors, and a tendency to social domination [18].

It was previously shown that mitochondria of the vital organs of LR and HR rats differ in both structural and basic functional parameters. Thus, the mitochondria of the cerebral cortex of HR rats are characterized by a denser packing of cristae and a more electron-dense matrix, smaller sizes, and higher concentrations of the respiratory chain complexes and the respiration substrate, succinate (i.e., they are functionally more active compared to mitochondria of LR rats) [16,25]. In contrast, in brain mitochondria of LR rats, the number of mitochondrial cristae is decreased, and this is consistent with the lower content of their respiratory electron carriers compared to mitochondria of HR rats. It was also found that in liver mitochondria of HR animals, the rate of the ATP-dependent potassium transport, which reflects the activity of the mitochondrial ATP-sensitive potassium channel, was increased [17].

Here, we have demonstrated that, in addition to the above structural–functional features, the mitochondria of the liver and heart of rats with originally different tolerance to hypoxia are distinguished in respect to the rate of influx of Ca^2+^ ions via MCUC. The liver and heart mitochondria of HR rats are characterized by a significant increase in the efficiency of Ca^2+^ uniport compared to the organelles of LR rats, with the difference being especially pronounced for the liver tissue. To elucidate the causes of these functional effects, we have examined structural changes in the Ca^2+^ transport system of mitochondria of LR and HR rats and revealed that these changes do occur in the macromolecular complex of the mitochondrial Ca^2+^ uniporter in the heart and liver tissues.

The MCUC is considered to consist of two transmembrane channel subunits: MCU and MCUb. MCU forms a highly selective Ca^2+^ channel, which transfers the ion across the inner mitochondrial membrane. MCUb is a dominant-negative MCU paralogue, and its overexpression impairs the ion-transporting function of the complex [13]. MCU and MCUb are associated with other subunits: MICU1-2, EMRE, and MCUR1, which act as regulators of the uniporter.

As follows from the data obtained, in HR animals, the contents of the pore-forming subunit MCU and the gate subunit MICU1 are substantially increased in liver mitochondria, whereas the level of the dominant-negative subunit, MCUb, is decreased in heart mitochondria. The data are confirmed by the results of real-time PCR analysis and indicate that these changes occur at the level of transcription. At the same time, there is almost no difference between liver and heart mitochondria of LR and HR animals in the content of the regulatory uniporter subunit EMRE and the expression of its gene.

It is known that the activity of MCUC is regulated by changes in the ratio of the Ca^2+^-conducting pore subunits MCU and MCUb [5,26,27]. Therefore, both an increase in the level of MCU and a decrease in the content of its paralogue MCUb, which we recorded in mitochondria of the liver and heart of HR animals, can be the cause of the increased rate of Ca^2+^ influx into the organelles. Based on the data obtained, we can conclude that the structural changes in the pore-forming subunits of MCUC in HR rats increase the efficiency of Ca^2+^ accumulation in the mitochondrial matrix and, as a result, might underlie the fine control of cytoplasmic free Ca^2+^ at low levels.

On the other hand, excessive accumulation of Ca^2+^ into mitochondria can result in the opening of MPT in the inner mitochondrial membrane. As a result, all transmembrane ionic gradients and the membrane potential collapse, and mitochondria swell, which leads to the rupture of their outer membrane and the release of proapoptotic proteins (cytochrome *c*, apoptosis-inducing factor, and others) from the organelles. This can facilitate the activation of the caspase cascade, DNA damage, and eventually cell death. The opening of the MPT pore is believed to be triggered by hypoxia and subsequent reoxygenation [3,14]. The data obtained in this work show that liver (but not heart) mitochondria of HR rats have a significantly higher Ca^2+^ capacity (i.e., are more resistant to the induction of the MPT pore than the organelles of LR rats). Similar results were partially obtained by us earlier [19]. In order to gain insight into the molecular mechanism of tolerance of liver mitochondria of HR rats to MPT, we assayed for levels of MPT-associated mitochondrial proteins: cyclophilin D, ATP synthase (α-subunit), and adenylate translocator ANT1 (Figure 4). Our results indicate that the levels of cyclophilin D and ANT1 in mitochondria of the liver of HR and LR rats are not distinguished. There is, however, a significant decrease in the content of the α-subunit of ATP synthase (a protein that supposedly forms the channel of the MPT pore) in liver mitochondria of HR rats. Thus, the increased tolerance of liver mitochondria of HR rats to MPT, as compared with mitochondria of LR rats, can be related to a lowered expression of the MPT pore-forming protein. This is in agreement with the earlier report that the changes in expression of ATP synthase had significant effects on the probability of MPT induction [28].

However, the question as to which subunit of this multisubunit complex is the central structural component of the MPT pore, or whether it is formed by MPT-specific conformation of dimers of ATP synthase, remains the province of further investigations.

It should be noted that the CRC index of mitochondria isolated from the heart of HR rats was slightly lower compared to that of LR rats, although this difference was not statistically significant. This trend toward a decline in the Ca^2+^ buffering capacity is likely explained by the fact that the content of cyclophilin D, a major regulatory protein of the MPT pore [14,29,30], in heart mitochondria of HR rats is increased (Figure 6). It is believed that VDAC1 is also involved in the formation of the MPT pore complex in mitochondria. In addition, this protein participates in the formation of MAM contacts, which are the main pathway of Ca^2+^ transfer from the endoplasmic reticulum to mitochondria [5,14]. So, the changes observed in the level of VDAC1 can also contribute to the tissue-specific modulation of mitochondrial Ca^2+^ handling and the MTP opening.

Based on the results obtained, one can assume that the increase in the rate of Ca^2+^ transport in mitochondria is not accompanied by a higher susceptibility of mitochondria to calcium overload and the induction of the MPT pore. As follows from our data, changes in the levels of the MPT-related proteins can play a crucial role in the regulation of pore formation in mitochondria. At the same time, the high rate of mitochondrial Ca^2+^ uptake may provide a more effective removal of excess Ca^2+^ ions from the cytosol under physiological conditions and hypoxia.

## 5. Conclusions

The results obtained point out that liver and heart mitochondria of HR animals exhibit functional and structural features in Ca^2+^ ion transport, which may be essential for proper mitochondrial function in hypoxia and contribute to the formation of adaptive signs to provide the development of cellular response to oxygen shortage. So, apart from the increased activity of the ATP-sensitive potassium channel and respiratory chain complexes, mitochondria of HR animals are characterized by the increased rate of uptake of Ca^2+^ ions. At the same time, compared to liver mitochondria of LR animals, the organelles of HR rats are more resistant to the opening of the Ca^2+^-dependent MPT pore. Taking into account the fact that the MPT pore opening is one of the key pathogenic events in oxygen deficiency, it can be supposed that these changes contribute to the molecular basis underlying cellular sensitivity to hypoxic injury.

## Figures and Tables

**Table 1:** List of gene-specific primers for RT-PCR analysis.

Gene	GenBank #	Sequence 5’-3’	Product Size (bp)
*MCU*	NM_001106398.1	F: GCCACCAAAGAGAGACCTCC R: GCTCAATGCACAGTGTGGTG	98
*MCUb*	XM_006224254.3	F: CGCCCCAGGTTTCAGGTATG R: GGCAGGGTGAGGGTTACAAA	136
*MICU1*	NM_199412.1	F: AGGACTTTGTGCGCTCCATA R: GTTCCTGGGCAATTTTCTTTCCA	105
*Slc25a4 (Ant1)*	NM_053515.1	F: TGCCAGACCCCAAGAATGTG R: GTACATAATATCAGCCCCTTTCCG	149
*Ppif*	NM_172243.1	F: GGTGCTGGAGTTAAAGGCAGATG R: TGATTGGTGAAGTCGCCAGC	150
*Atp5f1a*	NM_023093.1	F: GACAGACCGGGAAAACCTCG R: GGTGGACCGTTTCTGACCAA	125
*Vdac1*	NM_031353.1	F: AGGGCTACGGCTTTGGCTTA R: AAACGTCAGCCCATACTCGG	155
